# Supplementary material for: The Effect of the Synthetic Procedure of Acrylonitrile–Acrylic Acid Copolymers on Rheological Properties of Solutions and Features of Fiber Spinning
Source: Materials (Basel). 2020 Aug 5;13(16):3454. doi: 10.3390/ma13163454 (PMC7475841; doi:10.3390/ma13163454)
Supplement: Supplementary file 1 [file materials-13-03454-s001.pdf]

materials

MDPI

Supplementary Materials: The Effect of the Synthetic Procedure of Acrylonitrile–Acrylic Acid Copolymers on Rheological Properties of Solutions and Features of Fiber Spinning

Ivan Y. Skvortsov [1,*], Elena V. Chernikova [1,2], Valery G. Kulichikhin [1], Lydia A. Varfolomeeva [1], Mikhail S. Kuzin [1], Roman V. Toms [3] and Nikolay I. Prokopov [3]

[1] A.V. Topchiev Institute of Petrochemical Synthesis, Russian Academy of Sciences, Leninsky Av., 29, 119991 Moscow, Russia; chernikova_elena@mail.ru (E.V.C.); klch@ips.ac.ru (V.G.K.); varfolomeeva.lidia@mail.ru (L.A.V.); gevahka15@gmail.com (M.S.K.)

[2] Department of Chemistry, M.V. Lomonosov Moscow State University, Leninskiye Gory, 119991 Moscow, Russia

[3] M.V. Lomonosov Institute of Fine Chemical Technologies, Russian Technological University, Vernadsky Av., 86, 119991 Moscow, Russia; toms.roman@gmail.com (R.V.T.); prokopov@mitht.ru (N.I.P.)

* Correspondence: amber5@yandex.ru

The Nature of Difference of RAFT-Based and Conventional Copolymers of Acrylonitrile

The recent progress in the reversible–deactivation radical polymerization (RDRP) is based on the successful synthesis of macromolecules with complex architectures, in particular, block- and graft-copolymers of various topologies, as well as copolymers with controlled monomer sequence distribution. Reversible addition – fragmentation chain transfer (RAFT) polymerization being a type of RDRP, can be successfully used for the controlled synthesis of homo- and copolymers of acrylonitrile. The advantages of this approach over other RDRP techniques come from: 1) tolerance to functional groups of the reagents; 2) use of various types of radical initiation; 3) wide range of the polymerization temperatures; 4) synthesis of the polymers with narrow molecular weight distribution and desired MWs; 5) control over composition homogeneity in the case of copolymers; 6) low degree of branching due to suppression of chain transfer to polymer.

Briefly, this process is based on the multiple repeats of the acts of activation and deactivation of propagating radicals ($P_n^•$) via a reversible chain transfer reactions, which is caused by so-called RAFT agents of general structure ZC(=S)SR (where Z and R are stabilizing and leaving groups of different nature, typically aryl or alkyl):

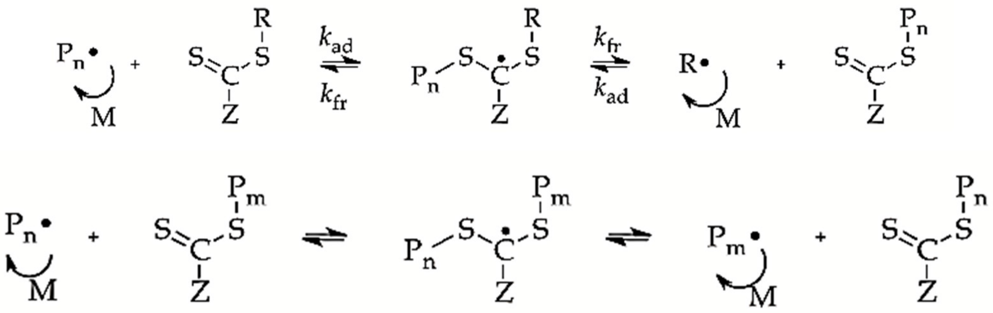

As a result, the macromolecules grow throughout the polymerization, while the irreversible termination of propagating radicals (typical for conventional radical polymerization) becomes negligible. The kinetic features of RAFT polymerization become similar to living anionic polymerization.

In the batch synthesis, the monomer unit sequence is determined by monomer reactivities (Fig. S1) and monomer feed. According to results presented in Figs. S2a, S3, S4a, S5, and S6a, the main difference between the samples A3 and B3 (Table S1) comes from MWDs. During polymerization of

sample A3 the MWD becomes broader, while M_n decreases due to decrease of monomer concentration. In contrast during polymerization of sample B3 the living features of polymerization are observed and Mn increases, while MWD is narrower. Both samples are characterized by random monomer distribution. According to our estimations the composition heterogeneity of sample A3 becomes noticeable after 80 % of monomer conversion, this comes from relatively low concentration of AA taken for the synthesis. While sample B3 is characterized by low composition heterogeneity due to living mechanism of polymerization.

In semi-batch polymerization the differences between the samples A2 and B2 includes composition heterogeneity along with MWDs. The similar trend in MWDs is observed (Figs. S2b, S3, S4b, and S5). Besides, in both samples the macromolecules formed up to middle conversions are pure homopolymers, PAN (Fig. S6). In the case of conventional radical polymerization (sample A2), the further increase of overall monomer conversion is accompanied by formation of new macromolecules comprising units of AA. The higher is monomer conversion, the more pronounced is composition heterogeneity. In contrast, in the case of RAFT polymerization (sample B2), the continuous activation of propagating radicals results in the formation of diblock copolymer PAN-*block*-poly(AN-*co*-AA). The higher is monomer conversion, the longer is the block of random copolymer of AN and AA. The living mechanism provides composition homogeneity of sample B2, but its significant heterogeneity along the macromolecule.

Finally, in continuous polymerization the situation should be similar to batch polymerization. Samples A1 and B1 differ mainly in MWDs (Figs. S2c, S3, S4c, and S5). Both samples are characterized by random monomer distribution. Comparing to batch polymerization, it may be noted that the monomer sequence is slightly different due to different instant concentration of AA in monomer feed (Fig. S6).

More details about polymer synthesis and characterization for AN and AA RAFT and conventional copolymerization using various methods of AA addition can be find in [1,2].

Table S1. The method of AA addition.

Method	Sample	The method of AA addition
Conventional radical copolymerization	A1	Continuous
	A2	Semi-batch
	A3	Batch
RAFT copolymerization	B1	Continuous
	B2	Semi-batch
	B3	Batch

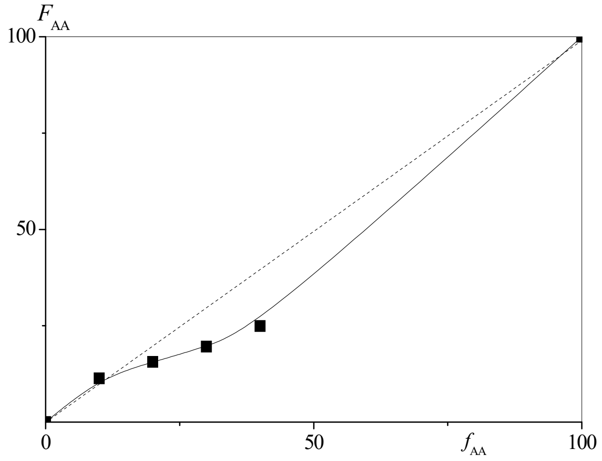

rAA = 0.82±0.26 и rAN = 0.69±0.05

Figure S1. Dependence of molar fraction of AA in copolymer (F_{AA}) from molar fraction AA in monomer feed (f_{AA}) for the copolymers formed at overall monomer conversions less than 10% in conventional radical copolymerization in DMSO, T = 55 °C.

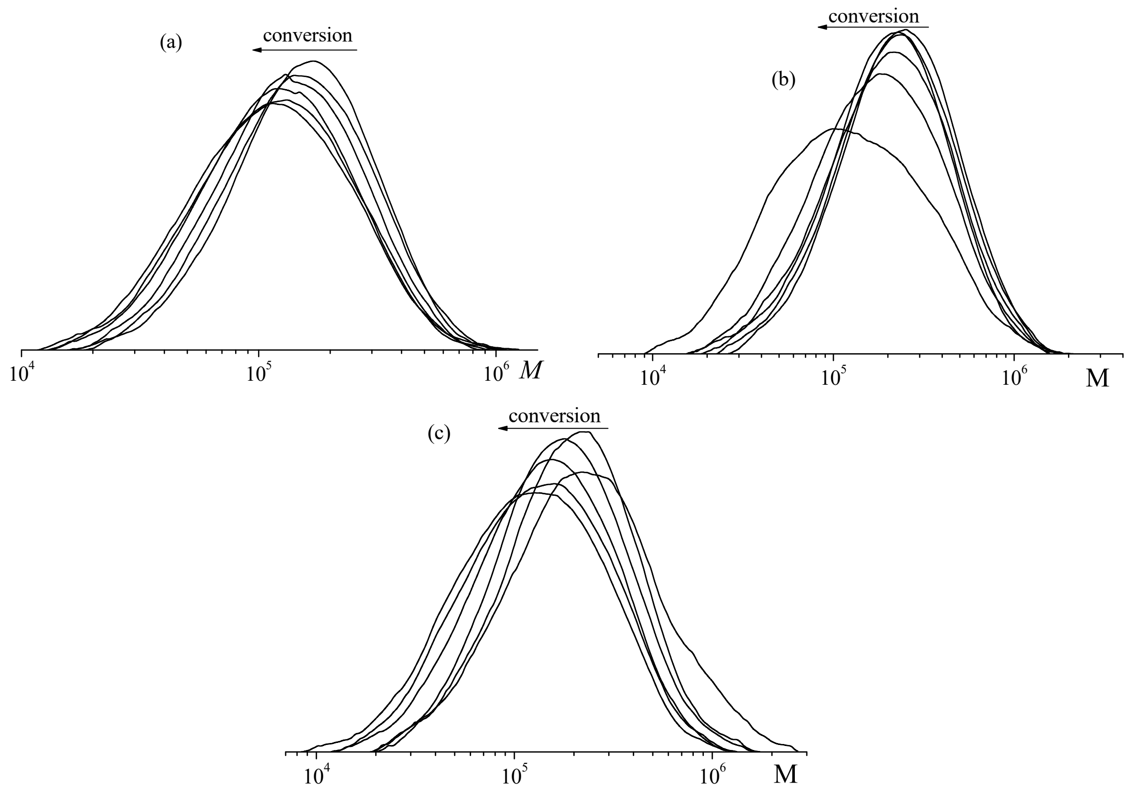

Figure S2. The SEC curves normalized by unit area for copolymers formed from monomer mixture of acrylonitrile and AA in batch (a), semi-batch (b) and continuous conventional radical copolymerization (c).

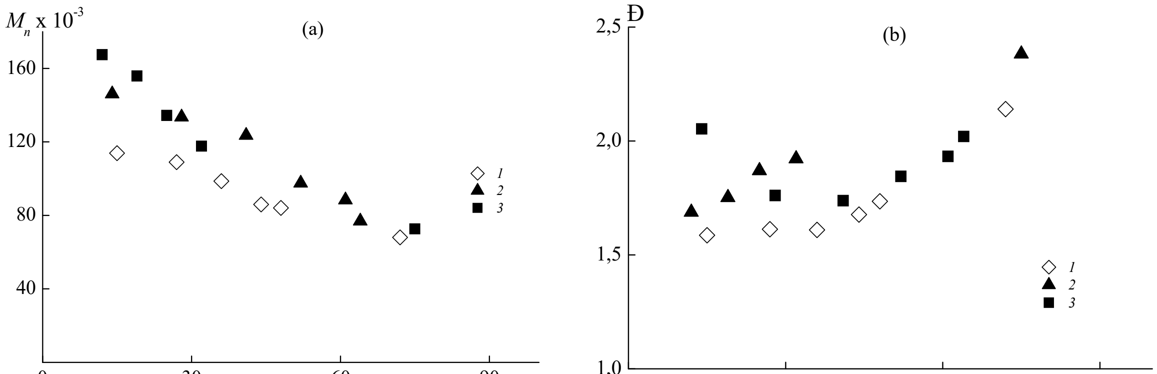

Figure S3. Dependence of M_n (a) and dispersity Đ (b) from overall monomer conversion for copolymers formed from monomer mixture of acrylonitrile and AA in batch (1), semi-batch (2) and continuous conventional radical copolymerization (3).

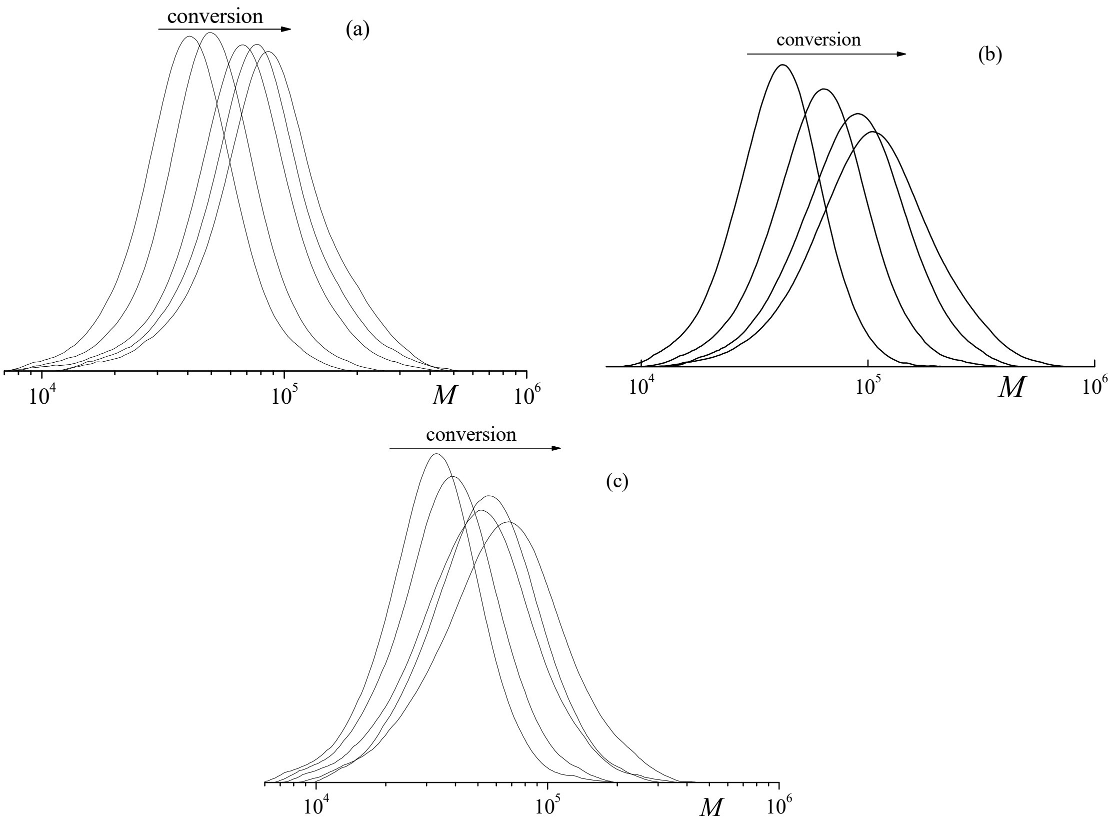

Figure S4. The SEC curves normalized by unit area for copolymers formed from monomer mixture of acrylonitrile and AA in batch (a), semi-batch (b) and continuous RAFT copolymerization (c).

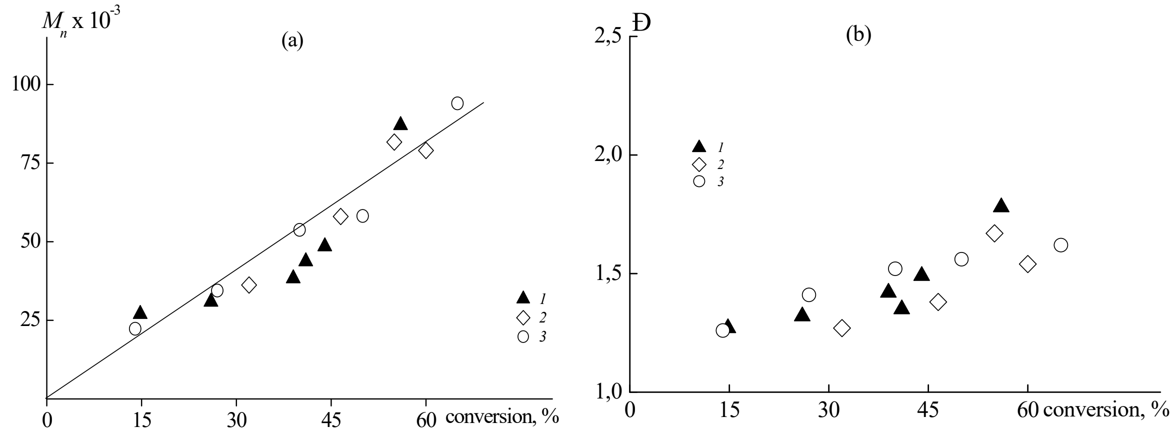

Figure S5. Dependence of M_n (a) and dispersity Đ (b) from overall monomer conversion for copolymers formed from monomer mixture of acrylonitrile and AA in batch (1), semi-batch (2) and continuous RAFT copolymerization (3).

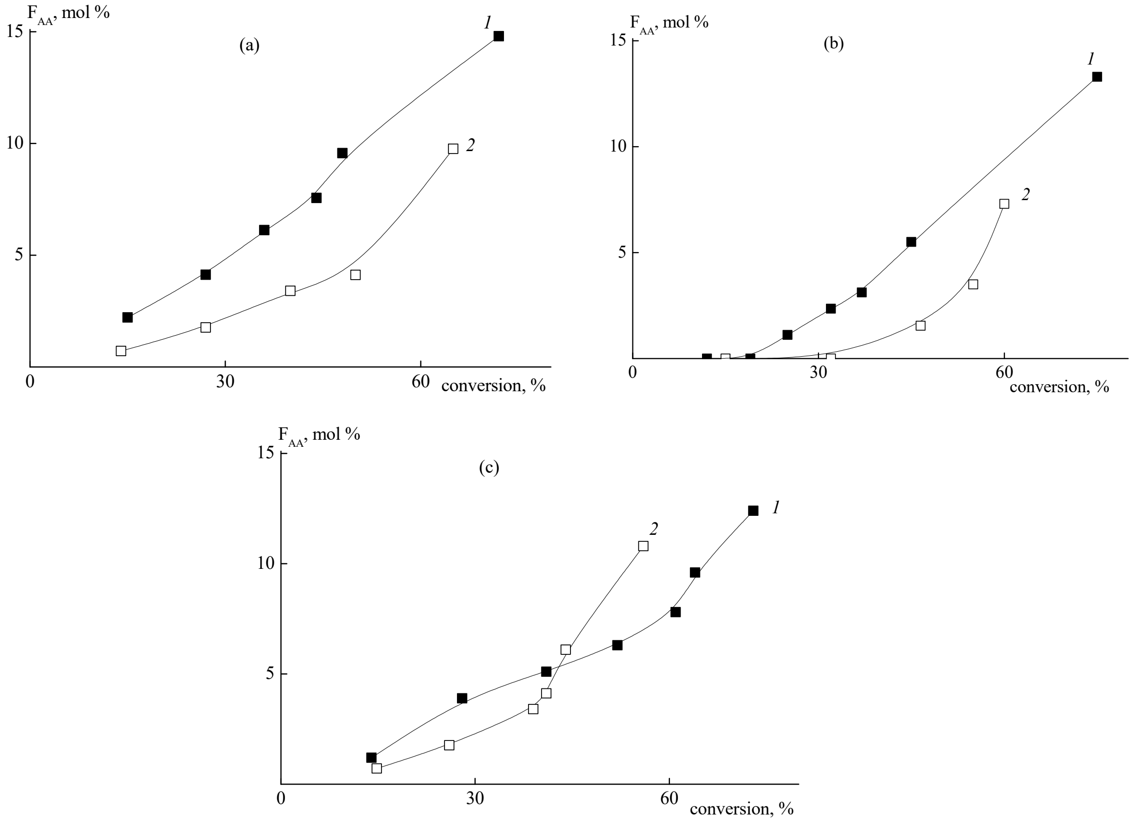

Figure S6. Dependence of molar fraction of AA in copolymer (F_{AA}) from overall monomer conversion for the copolymers formed in batch (a), semi-batch (b) and continuous copolymerization (c) via conventional radical mechanism (1) and RAFT mechanism (2).

Reference

1. Toms, R.V.; Balashov, M.S.; Shaova, A.A.; Gerval'd, A.Yu.; Prokopov, N.I., Plutalova, A.V.; Grebenkina, N.A.; Chernikova, E.V. Copolymers of Acrylonitrile and Acrylic Acid: Effect of Composition and Distribution of Chain Units on the Thermal Behavior of Copolymers. Polym. Sci. Ser. B, 2020, 62, 102-115, doi: 10.1134/S1560090420020086
2. Toms, R.V.; Balashov, M.S.; Gerval'd, A.Yu.; Prokopov, N.I.; Plutalova, A.V.; Berkovich, A.K.; Chernikova, E.V. The influence of the synthesis method on the properties of carbon fiber precursors based on copolymers of acrylonitrile and acrylic acid. Polym. Sci. Ser. B 2020, 62(6). (in press)

© 2020 by the authors. Submitted for possible open access publication under the terms and conditions of the Creative Commons Attribution (CC BY) license (http://creativecommons.org/licenses/by/4.0/).
